# Health Risks from Microplastics in Intravenous Infusions: Evidence from Italy, Spain, and Ecuador

**DOI:** 10.3390/toxics13070597

**Published:** 2025-07-16

**Authors:** Claudio Casella, Umberto Cornelli, Giuseppe Zanoni, Pablo Moncayo, Luis Ramos-Guerrero

**Affiliations:** 1 Department of Chemistry, University of Pavia, 27100 Pavia, Italy; icarocus@gmail.com (C.C.); gz@unipv.it (G.Z.); 2 School of Medicine, Loyola University, Chicago, IL 60153, USA; ucornelli@gmail.com; 3 Ingeniería Agroindustrial, Universidad de Las Américas (UDLA), Quito 170124, Ecuador; pablo.moncayo@udla.edu.ec; 4 Grupo de Investigación en Bio-Quimioinformática, Carrera de Ingeniería Agroindustrial, Facultad de Ingeniería y Ciencias Aplicadas, Universidad de Las Américas (UDLA), Quito 170124, Ecuador

**Keywords:** microplastics, intravenous infusion, exposure pathways, human health, disposable infusion tubes and syringes

## Abstract

The rising incidence of microplastics (MPs) is a possible health risk to humans. The present study aims to analyze the presence of MPs in intravenous (IV) infusions and compare MP concentrations from multiple brands. The IV solutions of 29 medical devices (IV-MDs) from seven brands commercialized in Ecuador, Spain, and Italy have been selected under specific consideration to carry out the study. The detection of MPs has been quite obvious in almost all of the samples from brands in the mentioned countries. MP concentrations ranged from 9 to 20 MPs/L in glass containers to 166–299 MPs/L in plastic bags, with the majority of fragments (63%) on fibres (37%) and more than 60% of particles less than 100 µm. Nine different types of MPs were identified in this study. High clinical risk was indicated by markings with >200 MPs/L. Nevertheless, the medium polymeric danger index (PHI) was 1.7. According to these outcomes, IV infusion is a direct exposure to MPs that may have harmful medical repercussions. It is imperative that MPs’ limitations be included in pharmacopoeic monographs and in vivo toxicological and epidemiological studies. The present study aims to analyze the presence of MPs in IV-MDs and compare MP concentrations from multiple IV-MD brands.

## 1. Introduction

Smaller than 5 mm plastic particles, called microplastics (MPs, 1–5000 µm), come in a variety of shapes and are released from plastic products, mainly additives, cosmetics, and other materials. Nanoplastics (NPs) are tiny plastic particles with a size range from 1 nm to <1 µm [1,2,3,4]. Over the past 20 years, a number of international organizations and scientific groups have devoted notable attention to these emerging pollutants and their significant impact on human health. The Plastics Europe study [5] claims that plastic production reached around 413.8 million tons globally in 2023 following the COVID-19 pandemic. Fossil fuels are believed to have accounted for 90.4% of global plastic output during that period.

To properly evaluate the possible health effects of MPs, it is essential to have a comprehensive understanding of the exposure pathways by which they infiltrate human tissues [6]. These particles are persistent, ubiquitous, and can potentially cross biological barriers, raising concern about their accumulation and toxicological effects in the body.

MPs have been found in a variety of frequently consumed foods and beverages, making regular dietary intake one of the main sources of microplastic exposure in humans. They can be continuously consumed and accumulated, as seen by their presence in foods like shellfish, salt, water, and even fruits and vegetables [7,8].

Initially, MPs were observed in human organs such as the intestines and lungs and in human stools [6,9]. More recent studies, however, have indicated the presence of MPs in the cardiovascular system, including blood vessels, veins, and the heart. The detected MPs were found to consist of a variety of polymers, including polyvinyl chloride (PVC), polystyrene (PS), polyethylene (PE), polyvinyl alcohol (PVA), polyethylene terephthalate (PET), expanded polystyrene (EPS), acrylonitrile butadiene styrene (ABS), polymethyl methacrylate (PMMA), polyurethane (PU), low-density polyethylene (LDPE), polyamide/nylon (PA), along with copolymers like Styrene-butadiene (SBR) and ethylene-vinyl acetate (EVA) [10,11,12,13]. However, their existence in human blood has garnered more significant attention [10,14]. As a primary internal transport system, the circulatory system plays a crucial role in delivering oxygen, nutrients, and hormones throughout the body. Consequently, MPs entering the bloodstream can be transported to various organs and tissues [15]. The mechanisms by which MPs enter the bloodstream are still not well understood despite the growing body of research on MPs in human health. According to common belief, MPs enter the human bloodstream through ingesting and inhalation, then pass biological barriers via processes including paracellular diffusion or endocytosis [16,17,18]. MPs may also enter the human body through intravenous medical devices (IV-MDs) such as surgical masks, gloves, syringes, and other single-use items composed of plastic or materials coated with plastic [11,19,20]. However, the direct entry of MPs into the bloodstream is particularly led by the use of plastic bottles containing IV-MDs. These are sterile combinations of drugs, aseptically mixed in liquid-filled bottles such as lactated Ringer’s solution, dextrose, normal saline, and several electrolytes for long-term treatment and concurrent drug administration in patient care [11,21,22].

In addition to their physical presence in human tissues, MPs can also serve as carriers for dangerous chemical additions, which makes them a serious health concern. Lead, cadmium, and mercury are examples of heavy metals; polycyclic aromatic hydrocarbons (PAHs); persistent organic pollutants (POPs), including dioxins and PCBs; and organophosphate esters (OPEs), which are employed as plasticizers and flame retardants [23,24]. These chemicals have the ability to leak from MPs into the body and interact with cellular systems, which may result in oxidative stress, inflammation, altered hormones, neurotoxicity, and possibly the development of cancer. MPs are more bioavailable and easier to convey to delicate organs including the brain and reproductive system because of their small size. MPs thus provide a double risk: chemical toxicity and physical incursion.

OPEs are chemical compounds generated from phosphoric acid. Plastics, PU foams, textiles, paints, coatings, and electronic devices all use them extensively as plasticizers and flame retardants. OPEs are readily released into the environment or human body when plastics age, break down, or are exposed to heat and light because they are not chemically bonded to the polymer matrix. These OPEs have the potential to be adsorbed or trapped on the surface or structure of MPs as they develop [23]. As a result, MPs may serve as OPE transport vectors, making it easier for the substances to enter the body through the mouth, nose, or skin. According to toxicological studies, exposure to OPEs has been associated with a number of negative implications, including neurotoxicity, endocrine disruption, toxicity to the liver and kidneys, and the risk of cancer (especially from chlorinated OPEs like Tris(1,3-dichloro-2-propyl)phosphate or TDCPP) [23,25,26]. Moreover, OPEs have been found in human tissues, blood, urine, and breast milk, suggesting that they have the ability to bioaccumulate and spread throughout the body [26,27].

Most IV-MDs, including fluid containers, infusion sets, and syringes, are made of plastic. MPs may be released during the production, storage, and use, potentially entering the bloodstream along with the medication [6,11,21,28]. MPs ranging in size from 4.0 to 148.0 µm have been found in samples from nine infusion bottles in recent studies. These findings indicate that MPs could enter the bloodstream directly through IV infusion [22,29,30]. Studies using micro-Fourier Transform Infrared Spectroscopy (µFTIR) detected 2.91 ± 3.91 particles/L during IV infusion. Another study confirmed 1.0 ± 0.7 µg/L of particles in infusion bags and demonstrated that tubing was the primary source of MPs during infusion [31,32]. Saline with a pump releases 0.90 MPs/mL into the bloodstream, but without a pump, the concentration increases to 1.57 particles/mL [29]. The presence of NPs in hypertonic solutions was alarming, with a mean concentration of 62.82 ± 72.38 MPs/L. MPs were also identified in three different types of infusion bottles, and 11.66% of all samples contained MPs [22,33].

These studies verified the potential danger of ingesting MPs/NPs through various infusion methods. For instance, MP fragments of PET and PP were emitted by PET bottles and PP containers exposed to sunlight for three months [34]. Previous studies, however, mostly focused on the presence of specific MD types without assessing brand differences or key variables. Thus, comprehensive studies on the characteristics, contributing factors, and risks of MPs entering the bloodstream via IV-MDs are still lacking. While ingestion and inhalation have long been considered the primary exposure routes for MPs, recent work from North America and Asia suggests that IV infusion provides a direct and previously under-recognized pathway into the circulatory system [6,10].

**Figure 1:**
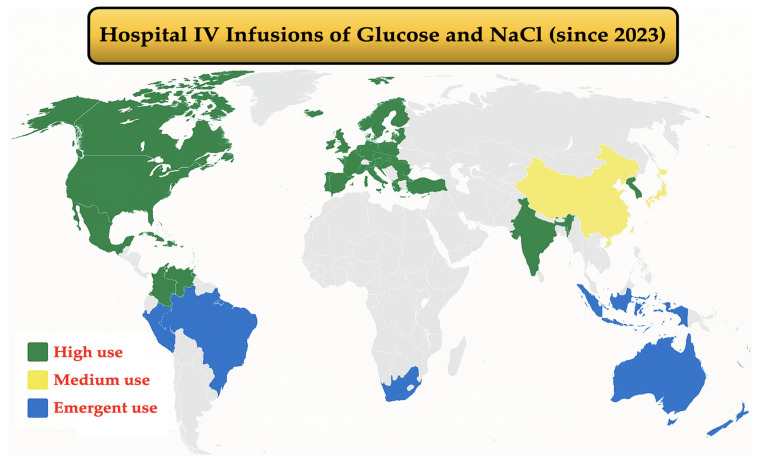
Main nations and areas with the highest hospital IV-MDs glucose and NaCl rates uses as of 2023 (source: Global Market Insights Inc., Selbyville, DE, USA).

The global IV-MDs market is projected to grow at a compound annual growth rate (CAGR) of 8% from 2024 to 2030, from USD 13.9 billion in 2024 to USD 22 billion by 2030, according to Grand View Research. Demand is driven by increasing incidence of diseases such as cancer, kidney disease, and gastrointestinal disorders, as well as rising rates of preterm births and malnutrition, particularly in neonatal and pediatric units (Figure 1).

The US dominated the IV-MDs market in 2023, valued at USD 5.1 billion, and is expected to grow to USD 10.6 billion by 2034. China follows as the second-largest market, forecasted to grow 7.9% annually between 2023 and 2030, reaching USD 2.2 billion. India leads global exports of glucose and NaCl infusions to countries like Ghana, Tanzania, and Uganda. Germany, the UK, and France are key European markets due to their strong healthcare infrastructure. Canada and Japan are projected to grow at annual rates of 6.3% and 5.6%, respectively. Kuwait is expected to have the highest CAGR between 2024 and 2030. South Africa and Brazil represent emerging markets with a growing IV therapy demand. In Europe, the IV glucose bag and NaCl solution market was valued at USD 812 million in 2023 and is expected to grow at a CAGR of 3.2%, reaching USD 1.011 billion by 2030. The NaCl IV-MDs market was estimated at USD 4.20 billion in 2025 and expected to reach USD 5.47 billion by 2030 (CAGR 5.45%), driven by a high prevalence of chronic diseases and ageing populations.

According to Mordor Intelligence’s Forecast 2024–2030, the European market was valued at USD 812 million in 2023 and is expected to grow at a CAGR of 3.2% to reach USD 1.011 billion in 2030. An ageing population and the increasing incidence of gastrointestinal disorders are the main causes of this rise, since they make glucose IV-MDs a crucial component of treatment. Table 1 provides details on the main segmentations.

The NaCl IV-MDs market was estimated at USD 4.20 billion in 2025 and is expected to grow at a CAGR of 5.45% to reach USD 5.47 billion in 2030 (source: Mordor Intelligence, Forecast 2024–2030) [35]. Due to the growing need for IV-MDs to treat a range of medical diseases, the global market for IV-MDs used in clinics and hospitals is expanding steadily. The main end-users of IV-MDs are hospitals and clinics. The high prevalence of chronic illnesses including cancer, neurological conditions, and gastrointestinal disorders, along with the ageing European population, are to blame for this recent increase. High incidence of chronic disorders, along with improvements and developments in patient convenience products, are expected to propel market expansion throughout the forecast period. By the end of 2023, there were over 279,260 new instances of cancer reported in Spain, of which 42,721 were colorectal cancer, 31,282 were lung cancer, and 21,694 were bladder cancer, according to statistics updated by the Spanish Network of Cancer Registries (REDECAN) in January 2023. Consequently, it is projected that the rising rate of cancer will raise demand for intravenous solutions and further propel market expansion. In Spain, over 279,260 new cancer cases were reported by the end of 2023. Italy had 407,240 new cases in 2022, with a slight female prevalence. Ecuador faces high rates of diseases requiring IV therapy, including leptospirosis and dengue (27,838 cases in 2023), and more than 60% of deaths are due to non-communicable diseases, mainly cardiovascular conditions and diabetes. Unlike Italy and Spain, Ecuador lacks current data on MP-related health risks. Critically ill patients face prolonged exposure to MPs due to the absence of monitoring systems. The most common cancers in Ecuador—breast, prostate, colorectal, stomach, thyroid, cervical, lymphoma, leukemia, lung, and liver—require IV chemotherapy, which increases potential MP exposure. Without MP contamination data in these MDs, Ecuador cannot fully assess health risks in cancer treatment settings. The main types of cancer in Ecuador, Spain, and Italy are illustrated in Figure S1.

According to estimates, Italy’s cancer incidence rate in 2022 was 4% higher for women than the EU average but 2% lower for males. Age-standardized cancer mortality decreased by 15% between 2011 and 2021, surpassing the EU average fall of 12%, and was 6% lower than the EU average. Italy had around a 6% higher 5-year cancer frequency than the EU standard in 2022. In 2022, there were 407,240 new cases of cancer in Italy, with 194,148 occurring in women (48%) and 213,092 occurring in men (52%) according to the Joint Research Center’s European Cancer Information System (ECIS) [36]. In 2022, lung, prostate, colorectal, and breast cancers collectively accounted for almost half of all cancer cases. With an 18% incidence rate, prostate cancer was the most common cancer in men, followed by colorectal (14%) and lung (13%) malignancies. One of the most prevalent cancers in women was breast cancer (31%), which was followed by colorectal (12%), lung (8%), and uterine (5%) (source: Country Cancer Profile 2025).

Ecuador has a high prevalence of illnesses that need IV treatment, like leptospirosis in the Amazon region, where fluid therapy is essential to preventing hypovolemic shock, and dengue, which had 27,838 cases in 2023, according to the Undersecretary of Surveillance, Prevention, and Health Control National Directorate of Epidemiological Surveillance (https://www.salud.gob.ec/direccion-nacional-de-vigilancia-epidemiologica (accessed on 25 March 2025)). More than 60% of deaths in Ecuador are caused by non-communicable diseases, mostly cardiovascular (34.9% of deaths) and diabetes mellitus, which often results in acute complications that require IV fluids [37]. In contrast to Italy and Spain, where research on effect of the MPs on human health is already reported, Ecuador lacks current information on this risk [38]. Patients with critical conditions are at a higher risk of prolonged exposure to MPs and their harmful effects on essential organs due to this lack of information, which makes it difficult to create surveillance and control measures for IV-MDs. Breast cancer, prostate cancer, colorectal cancer, stomach cancer, thyroid cancer, cervical cancer, non-Hodgkin lymphoma, leukemia, lung cancer, and liver cancer are among the ten most prevalent cancer types in Ecuador, all of which need treatment plans that involve IV chemotherapy (IV infusion) [39]. IV systemic chemotherapy is commonly used for all of these cancers, exposing patients to possible pollutants (i.e., MPs) from the plastic in the bags and Luer-locks of infusion sets. Due to the absence of data on MP contamination in these MDs, Ecuador is unable to assess the risk to human health posed to cancer patients.

The present study is the first to systematically compare MP concentrations between different IV-MD brands currently on the market in three countries—two in Europe (Italy and Spain) and one in South America (Ecuador), despite earlier research reporting the presence of MPs in IV-MDs. The aim of this comparative study is to assess possible variations brought about by elements including hospital-level handling, device packing materials, and storage settings (such as controlling temperature and humidity). The study specifically contrasts MPs in IV-MDs placed in glass and plastic containers. In all, 29 IV-MDs from seven distinct brands of IV solutions were analyzed (Table S1). The presence of built-in filters and gadget characteristics were considered, along with the brand and usage conditions. The results of the experiment and the data analysis were used to perform a risk assessment for human exposure. The lack of current data from Ecuador, in particular, indicates a significant global gap in IV-MD monitoring and quality management, and the study emphasizes how vital it is to set up surveillance systems in every country to guarantee patient safety.

## 2. Materials and Methods

### 2.1. Sample Collection

From specialized retailers, twenty-nine IV-MD items from seven different brands were acquired. For the IV-MDs that were bought in Italy, there were three that contained 0.9% NaCl and three that included 5.0% glucose. A single brand is used by all six IV-MDs (Brand 1). Brand 2 is the original manufacturer of the three IV-MDs containing 0.9% NaCl and three IV-MDs containing 5.0% glucose that were bought in Spain. Brand 3 provides the last 3 IV-MDs of 5.0% glucose and 0.9% NaCl. The 11 IV-MDs that were purchased in Ecuador are all 0.9% NaCl solution. Brand 4 has three IV-MDs, Brand 5 has two, Brand 6 has three, and Brand 7 has the final three. Every feature of the IV-MDs analyzed in the present study is illustrated in Table S1. Every sample was acquired from March to April of 2025. The worldwide, European, and South American IV-MD market profiles were examined in order to gain a better understanding of the potential negative impacts of MPs in these MDs.

### 2.2. IV-MDs’ Pre-Treatment

To prevent contamination from metal particles or other kinds of contaminants, all reagents and bifiltered distilled water were filtered using a glass microfibre filter (0.7 μm pore size, Whatman, Florham Park, NJ, USA). Within the vacuum cabinet, the full volume of every IV-MD was vacuum filtered. To recover any liquid that could have remained in each IV-MD container and to prevent bias in the identification and quantification of the metal particles included in each IV-MD, bifiltered distilled water was utilized after all of the IV-MDs had been deposited on the filter. All tests were conducted in triplicate for each type of IV-MD (n = 3). Since the separation of MPs in closed pharmaceutical systems, like IV-MD devices, has few documented precedents, we consider this protocol as an original and pertinent methodological contribution that is intended to guarantee efficient recovery with little interference from background material [40].

### 2.3. Microplastic Analysis

A semi-automatic stereomicroscope (Leica M205FA, Leica Microsystems CMS GmbH, Wetzlar, Germany) and a high-resolution colour digital camera (Leica DFC310FX; 1.4 Mpixel, CCD, Leica Microsystems CMS GmbH, Wetzlar, Germany) were used to count the MPs in filters. Furthermore, the MP fragment and MP fibre diameters were estimated using the Confocal UniOvi ImageJ 2025, programme. Scanning Electron Microscopy (SEM) analysis was used to assess the morphology of the MPs found in the IV-MDs (JEOL-6610LV with microanalysis, Tokyo, Japan). The scanning electron microscope had tungsten filament electron guns, a maximum resolution of 3.0 nm, and an operating voltage range of 0.5 to 30 kV. The range of magnification was 5× to 50,000×. It used high-vacuum modes for samples with the highest resolution and low-vacuum modes for wet or non-conductive surfaces. It used backscattered secondary (composition, topography, and shadowing) electron detectors. It had a 5-axis asynchronous mechanical eucentric stage that could handle samples up to 20 cm in diameter despite the eucentric tilt and rotation. The SEM was fully automated, operated on a single PC, and automatically saved images in the BMP, TIFF, or JPG formats. A µ-FTIR spectrophotometer (Perkin Elmer Spotlight 200i FTIR spectrophotometer, Springfield, IL, USA) from the Autonomous University of Madrid (UAM) Molecular Spectroscopy Unit was used to determine the chemical composition of the MPs. KBr pellets, which were transparent to infrared radiation, were used to support MPs for transmission analysis. The results of an automatic examination of the generated infrared spectra were compared with a spectral database, which is stored in the previously mentioned apparatus and contains around 36,000 spectra of different compounds. The spectral range (550–4000 cm^−1^), resolution (16 cm^−1^), number of scans (30), and infrared beam aperture (20 × 100 microns for MP fibres and 50 × 50 microns for MP fragments) were the measurement parameters used to study the MPs. The spectrophotometer of more than 36,000 substances, including organic and inorganic compounds, polymers, fibres, cosmetics and derivatives, solvents, drugs, etc., was used to compare the results. The micrometric samples were examined using a Euromex Edu Blue magnifying glass (Duiven, The Netherlands) with 20× and 40× magnification after the researcher obtained the sample count for each plate and the approximate size, shape, and colour of each plate (data collected prior to analysis). Upon visual identification, the samples were manually placed on the surface of a produced KBr pellet (transparent in the mid-infrared region) and then placed in a designated sample holder of the infrared microscope (Perkin Elmer, Springfield, IL, USA). A magnifying lens was always utilized for this important task as a visual aid. The micrometric samples were subsequently handled using a tungsten needle tip or absorbent paper tips, which are mainly used in dentistry but are perfect for microfibres with an electrostatic charge. Each plate sample was placed on the surface of the KBr pellet, and the sample holder was then transferred to the infrared microscopy stage, where each micrometric sample was inspected under a microscope. The micrometric samples were analyzed with infrared after the analysis process was configured with the previously mentioned measurement settings. The MPs are identified by comparing the collected spectra with the reference spectra recorded in the Spectrum database of the measurement instrument.

### 2.4. Quality Assurance and Quality Control (QA/QC)

The quality assurance and control (QA/QC) procedures were carried out utilizing techniques outlined by various authors [3,41,42], from MP sampling to quantification. Important QA/QC procedures included filtering chemical reagents before use, eliminating polymeric compounds in the lab, and using glass microfibre filters (0.7 µm pore size). Good field and laboratory practices (GLP) were employed throughout the sampling and analysis process to reduce the secondary contamination from MPs that were discovered in the air, on surfaces, and ultimately on the equipment. Consequently, the sampling and processing samples were made with as little plastic as possible; when this was not feasible, procedural blanks were utilized. It was thought that when Falcon tubes and plastic bottles were used for sample treatment and sampling, a specific quantity of MPs might be released. To ensure that the analyses would not be impacted, control trials were conducted in both scenarios. Falcon tubes and plastic bottles released MP at mean concentrations of 0.5 ± 0.2 MPs/L and 0.4 ± 0.2 MPs/L, respectively. According to previous studies, these contributions were deemed negligible because they made up less than 1% of the total MP concentration used in each experiment [3]. Finally, each analysis and experiment were conducted in triplicate. Every IV-MD sample filtration technique was carried out in a certified laminar flow cabinet under close monitoring. To avoid contaminating samples, this particular type of cabinet offers a clean air working environment that satisfies ISO 5 standards. It has a unidirectional flow of sterile, particle-free air. This strategy is commonly used as a component of MP contamination control techniques. Apart from operating in a laminar flow, the subsequent safety measures were observed:

- Wearing face masks, fibre-free lab coats, and nitrile gloves that have been previously cleaned to stop the operator or clothing from producing particles. Before each use, it is advised that all lab equipment, such as forceps, funnels, and filters, be thoroughly cleaned with Type I ultrapure water. Laminar flow should then be used to dry any remaining residue.
- Blank controls: To assess any potential unintentional contamination throughout the process, procedural blanks, or control filters devoid of samples, were processed in conjunction with the experimental samples.
- To reduce their exposure to the environment, the filters should always be kept in closed or covered systems. A high level of confidence is provided by these approaches that the MPs found in the samples are solely from the IV-MDs under investigation and are not the result of contamination from outside sources or experimental artefacts.

### 2.5. Statistical Analysis

The mean and standard deviation were computed using three replicates for each group. Data analysis was performed using SPSS (version 24.0) for Windows (IBM, Armonk, NY, USA). The Tuckey HSD post hoc test was computed after an ANOVA was completed to ascertain the differences between the groups. The MP concentration was displayed using the average ± SD. The alpha value for statistical significance was chosen at 0.05 (*p* < 0.05). All tests were conducted in triplicate for each type of device (n = 3). In order to determine whether results are statistically significant, the alpha significance level was set at 0.05 (*p* < 0.05), which is a common criterion in scientific research. This type of experimental study, which aims to strike a balance between statistical rigour and available resources, typically uses three replicates per group, which is sufficient for identifying significant changes when there are obvious differences between groups. This process guarantees that the findings are solid and trustworthy, bolstering the study’s conclusions.

## 3. Results and Discussion

### 3.1. Comparative MP Analysis Among All IV-MDs Brands

As previously reported in the literature, this study concentrated on particular IV-MDs (bags, syringes, infusion tubes, etc.), [6,11,30,33]. This study differed significantly in that we examined two kinds of IV solutions (NaCl 0.9% and glucose 5%); glass and plastic containers of different brands from three distinct countries (Ecuador, Spain, and Italy) were analyzed.

**Figure 2:**
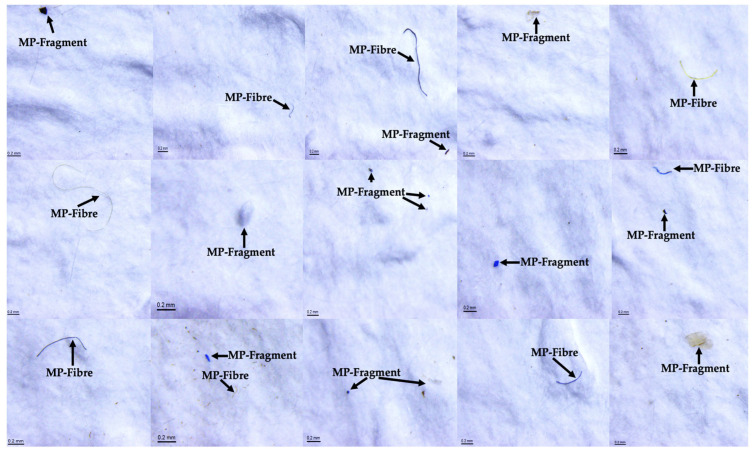
Stereomicroscope images of MPs found in IV-MD samples.

Examples of MPs found in all IV-MD samples are presented in Figure 2. MPs were detected in all 29 IV-MDs selected for the current study. Table S2 provides the characterization of the MPs identified with the aim to determine their size, shape, and concentration in each IV-MD.

In general, IV infusions containing 5% glucose had a slightly higher percentage of MP fragments (up to 68%) than fibres, according to a comparison of the data obtained for each IV-MD. The ranges are fairly comparable for the 0.9% NaCl solutions, with a slight bias toward MP fragments in essentially all of the samples. When comparing the data for each brand, it can be gathered that Brands 2 and 3 from Spain and Brands 4 and 7 from Ecuador exhibit more variation in MP concentration (MPs/L) and in the percentage of MP fragments against MP fibres. Small MPs (1–50 µm, roughly 50–56%) are most abundant in Brands 4 and 5 (Ecuador); however, Brands 6 and 7 move significantly closer to the medium range (51–100 µm). The MP range of 1 to 50 µm has the highest percentage (between 20% and 56%), according to the MP sizes for each IV-MD. The second most prevalent group of MPs (about 16–28%) resides in the 51–100 µm size range, with Brands 4 and 5 (Ecuador) at the lower end (~21%). Data for MPs with dimensions of 101–250 µm are more dispersed; Spain (i.e., Brand 2, NaCl) achieves 39%, whereas other brands range between 10% and 24%. Lastly, the contribution for MPs larger than 250 µm generally amounts to minimal (<12%), and in certain situations, it is absent from specific brands (i.e., Brand 2, NaCl 0.9%, Spain, and Brand 7, NaCl 0.9%, Ecuador) (Table S2). In conclusion, when comparing 0.9% NaCl IV-MDs with 5% glucose IV-MDs, glucose tends to result in a small relative rise in MP fragments relative to MP fibres; nevertheless, in practical terms, the differences are minimal (between 5 and 10 percentage points). All brands behave similarly; nearly all concentrate over 60% of their MPs in particles smaller than 100 µm, and only a small percentage exceed 250 µm. Regarding the median size (101–250 µm) and the MP fragment concentration, there is a wider dispersion in Ecuador (Brand 6) and Spain (Brands 2 and 3).

Glass IV-MDs have less MPs, as may be expected. For instance, Italian IV-MDs have extremely low counts, usually ranging from 9 to 19 MPs/L (average value ≈14 MPs/L). In glass bottles (i.e., NaCl 0.9%), Spain similarly displayed low values (such 13–20 MPs/L); but in plastic bags, MP levels rise to 211–240 MPs/L. Ecuador’s IV-MD samples (all in plastic bags) had an average of approximately 215 MPs/L and ranged from 168 to 299 MPs/L. The lowest MP concentration values were found in Brand 1 (Italy, glass bottles), which ranged from 11 to 16 MPs/L. The next lowest MP concentration values were found in the glass bottles of Brand 2 (Spain, 13–20 MPs/L). Brand 5 (plastic bags from Ecuador) had the greatest MP concentration, reaching 299 MPs/L. Ecuadorian Brands 4 and 7 typically have average MP concentrations above 200 MPs/L (Table S2). In fact, one important consideration is the type of the IV-MD container: plastic bags may increase MP emission, whereas glass reduces it. Due to surface abrasion, microscopic scratches, and material stress, MPs shed from polymers are typically very small, with an average size of less than 100 µm for all brands in all three countries. As a result, MP fibres and MP fragments are generated, generally less than 100 µm. Particularly, in situations involving movement or light storage, MPs have a tendency to split upon release instead of forming large particles.

**Figure 3:**
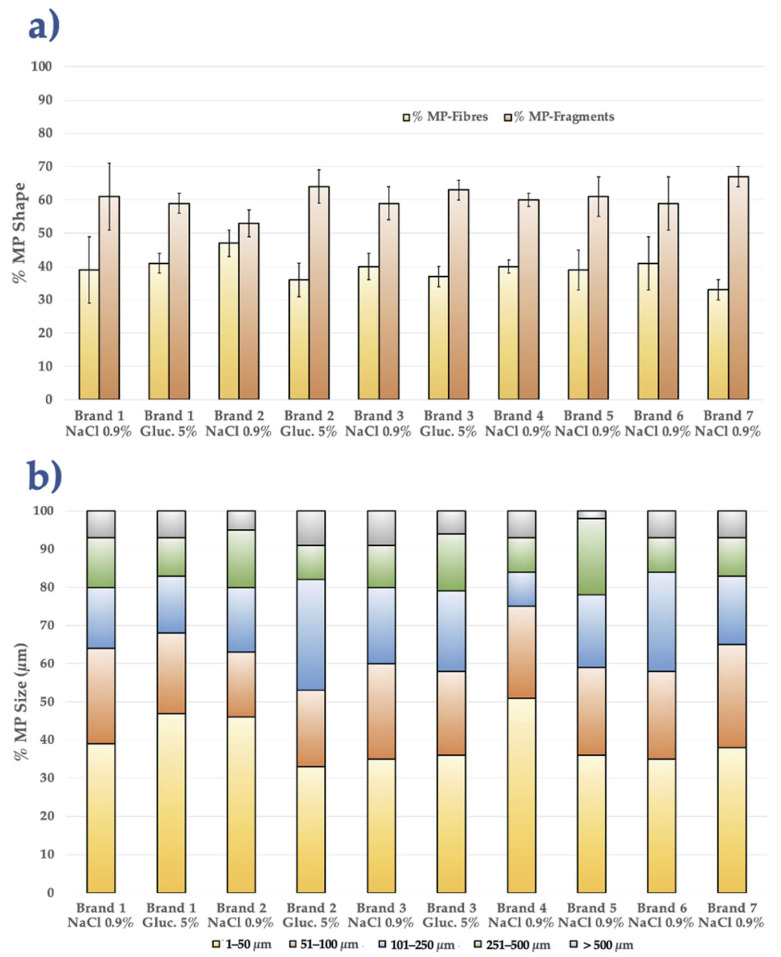
% MP fibres vs. % MP fragments “(**a**)” and % MPs in each size range “(**b**)” for all IV-MDs analyzed (type and brand).

Figure 3 represents the % of MP fibres compared to the % of MP fragments and the percentage of MPs in each size range for every combination of IV-MD solution type and brand. When comparing the current findings of the present study with those found in the literature, it is evident that all of the studies define sizes with a majority component of less than 100 µm. Only a few studies showed majority values with a range of MPs > 500 µm [11,31]. The MP concentrations (19–299 MPs/L) observed in the present study are in the mid-to-high range of those reported in previous studies. The lowest results were less than 5 MPs/L [6,31]. The highest results ranged from 5450 to 7500 MPs/L [20,28,29,31]. Glass container values (9–20 MPs/L) are comparable to the majority of studies [6,21,31]; nevertheless, plastic bag values (168–299 MPs/L) are comparable to [11].

The distribution of MP fragments > MP fibres is consistent with the majority of overall patterns in the literature, particularly in areas with plastic and bags [6,21,28,29,31,43]. MP films were only discovered as a third group of MP shapes in one study [44].

The tropical climate of Ecuador, with high temperatures and UV radiation that hasten the breakdown of plastic polymers and produce more MP fragments and MP fibres, may be the root cause of the highest MP concentration in the country in IV-MDs. Furthermore, the high humidity may be the cause, as this promotes the internal abrasion of plastic packing. Longer and more complex supply chains, which import and ship products from other countries and put plastic bags under more handling and mechanical strain, could be another culprit. Furthermore, cross-contamination and the environmental burden may be exacerbated by its closeness to MP-rich marine currents (Pacific currents) that transport debris made of plastic [45]. Conversely, countries with Mediterranean climates—such as Italy and Spain—have more mild temperatures, less constant exposure to strong UV rays, and regulated humidity levels, all of which often reduce the amount of plastic that degrades during storage and transit. Along with regulations regarding the environment that help prevent MP contamination, these countries also tend to have shorter supply chains and use standardized processes to minimize the mechanical stress on products. Regulations and handling and storage procedures should be tailored to the local context, taking into consideration the meteorological and logistical variations that contribute to the observed variability in MP concentrations in IV-MD between countries [46]. In order to guarantee the highest level of safety and quality in intravenous medical devices, Europe has a strong, thorough, and extensive regulatory framework. Ecuador is currently revising and upgrading its legislation to meet similar standards. In addition to environmental and logistical considerations, these regulatory variations have an impact on how devices are managed and quality controlled in each area.

**Figure 4:**
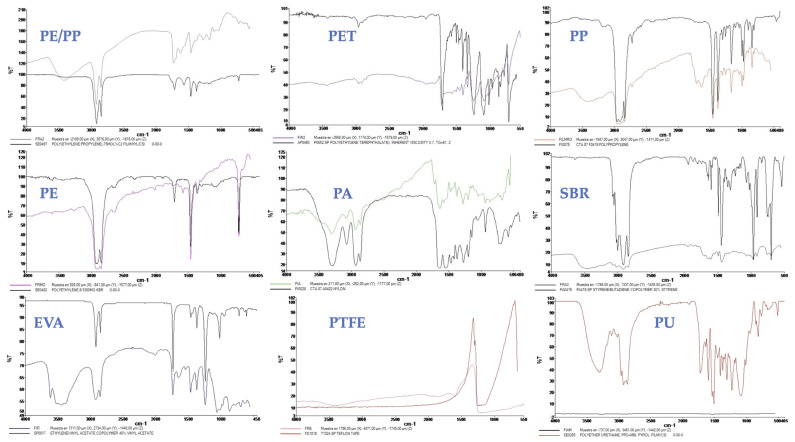
Main MPs found in all IV-MDs and tubes/catheters analyzed in the present study.

The most common MPs found in plastic bags, PP (36%), PE (21%), PA (12%), PU (9%), and PET (4%), were the polymers most commonly found in IV-MD bags. SBR (8%), EVA (5%), and PTFE (5%) were further MPs identified for technical components (i.e., catheters, infusion tubes, syringes, etc.). The most prevalent MP types were fibres (37%) and fragments (63%); these outcomes are consistent with other research reported in the literature [6,11,44]. Based on µFTIR analysis, the MPs identified in the present study are shown mainly in Figure 4.

- PP: It is the polymer used to make bags and some barrier layers. It can also occasionally be found as a copolymer with PE (PE/PP). Only PP particles (1–62 µm) were found in a trial including two brands of saline bags, with an estimated 750 MPs per bag [20]. It is utilized for IV bags, particularly in items that require autoclaving (at pressure and temperature) for sterilization. NPs < 50 nm (≈2.1 × 10^−4^ NPs/mL) and particles of 2–10 µm (≈216 MPs/mL) were discovered as a copolymer [47]. It may be present due to fragments produced during production or assembly, as well as mechanical wear (component friction).
- PE/PP: It is utilized in IV bags because it makes it simple to close the bags during production, which is essential for preserving sterility. In clinical settings, PE/PP copolymer-based infusion bags are the preferred option because they guarantee IV treatment administration that is both safe and effective. They may be present as a result of fragments released during production or assembly, or mechanical wear, which is the result of friction between components.
- PE: Specifically, low-density polyethylene (LDPE). It is a component of syringes, caps, connections, and the inner layers of some multi-layer IV bags. This polymer is flexible, has strong chemical resistance, and—most importantly—works well with drugs. Both mechanical wear (component friction) and fragments discharged during production or assembly may be the cause of its presence.
- SBR: Infusion tubes devoid of Di(2-ethylhexyl)phthalate (DEHP) are made with it as a contemporary substitute for PVC in PVC-free products. In addition to being soft and flexible, this polymer is also very biocompatible and is devoid of plasticizers. Due to their elasticity and resilience to chemicals, rubber components are used in vial or bag stoppers or septa, as well as in connections or other portions of the infusion system, such as adapters, tubes, or connectors. It is employed as part of secondary packaging materials. Short fibres or irregular fragments are produced when SBR is used as a gasket or splice and is abrased by contact against hard materials (plastic or metal). Micrograms are more likely to be released during the sterilizing phase in SBR if radiation (such as gamma or electron radiation) is used to destroy the polymeric network.
- PU: Filter membranes and seals, as well as some high-performance flexible tubing and IV catheters, use it. It is highly elastic and biocompatible. PU develops surface cracks in tubing bends and places that are bent repeatedly; these cracks can separate as MPs that measure 20 to 100 µm (such as flex fatigue). Long-term exposure to disinfectants or solvent solutions (such as alcohols or H_2_O_2_) can also break down urethane bonds, causing MP fragments to be released.
- PTFE: Internal valve components, coatings for extremely reactive or sensitive drug systems, and occasionally specialized catheters are among its uses. It is employed due to its superior heat resistance and excellent chemical inertness. Additionally, its surface is non-stick. It is also used in technical or stiff components of infusion sets that need to be precise, transparent, stiff, or resistant to chemicals. The PTFE coating may deteriorate with repeated fluid flow and connecting device use, releasing micronized flakes or layers. Microcracks in the film can be caused by abrupt changes in temperature or pressure, and as these breaks spread, fragments are released.
- EVA: It is the perfect material for bags that need to endure handling and transportation because it is flexible and remains intact under stress. Medical fluids including saline solutions, glucose, and prescription drugs can all be used with it. It offers a moderate barrier to oxygen. Due to the absence of plasticizers (i.e., phthalates), which can migrate into the IV fluid and are frequently hazardous, it is utilized as a substitute for PVC. Very thin sheets or flakes may be released when MP-EVA cracks or peels in places where it is repeatedly folded, such as bends in soft tubing. Vinyl bonds in EVA can be broken by this phase if sterilized with radiation (such as gamma or electron radiation), creating particles that range in size from 10 to 50 µm.
- PA: It is applied as an outer or intermediate layer to increase the resistance of the bag to high temperatures and punctures (such as those that occur during autoclaving procedures). For oxidation-sensitive solutions, its high oxygen barrier is essential. During filling, storing, and using the bag, it helps keep its integrity and shape. Many IV bags are multilayered structures composed of combinations including PA/EVA or occasionally PP/PA/EVA. Each layer has a distinct purpose, such as PA (intermediate or outer layer, which provides strength and acts as a barrier) and EVA (inner layer, which is in touch with the solution). This combination guarantees the sterility and longevity of the bag. It prolongs the stability of drugs or liquids by not releasing known pollutants. They can be released after several cycles of insertion and flexing.

**Figure 5:**
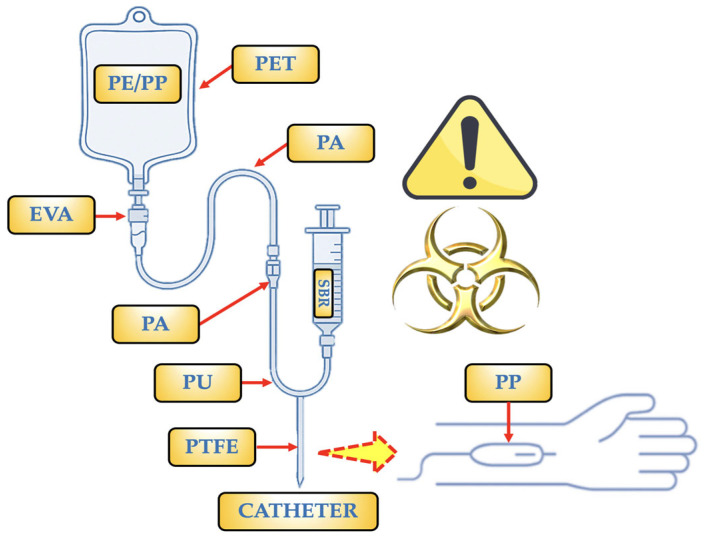
Sources of MPs analyzed in IV-MDs samples.

The origin of each MP found in the IV-MDs is represented in Figure 5 based on these considerations. According to the data collected for this study, MPs are thought to be the result of material separation or mechanical deterioration during fluid delivery. The detection of MPs in all IV-MDs—aside from the glass bottles—indicates that contamination occurs often during medical administration. IV-MDs emit particles as a result of wear, mechanical contact, or fluid interaction, as evidenced by the presence of fibres and fragments. Different medical devices (catheters, bags, and tubes) and their component materials have different MP concentrations and types. According to the data analysis, infusion tubes are more likely to become contaminated with MPs since they come into close contact with the fluid flow and include a higher variety of MP sizes. Interestingly, there were no detectable MPs in glass bottles used for IV infusions, in contrast to plastic bottles that leak fibres and fragments (PP, PE) [22]. Hospitals frequently use PP and PE containers because they are inexpensive and lightweight, however they are a major source of MP contamination. These may discharge particles into the fluid while being stored or infused.

Some of these polymers have the ability to react or adsorb specific pharmaceuticals (i.e., lipophilic drugs stick to PVC). Thus, lipid solutions, parenteral nutrition, and sensitive drugs are treated with EVA and PP. The numerous qualities and characteristics of every MP determined in the IV-MDs analyzed in the present study are summarized in Table S3. In general, sterilizing procedures (such as heat or radiation) can produce and release all MPs by breaking down the polymeric surface and creating detachable microfissures. Furthermore, interior surface abrasion results from therapeutic handling that involves frequent connections and disconnections. Due to surface cracking caused by temperature variations or friction during packaging, storage and transit conditions can also be sources of MP release. The use of alternative elastomers in gaskets and seals, such as medical silicone, which gives a reduced release of MP fragments, may be one mitigating strategy. Degradation-indicating changes in the “spectral signature” can be detected by process control, which includes in-line FTIR monitoring during tube and gasket production. To prevent recurring stress zones, reduce the bend angles in EVA hoses. To verify release limitations, use pulsatile flow tests in conjunction with serial filtering and MP-EVA/MP-SBR particle quantification.

**Figure 6:**
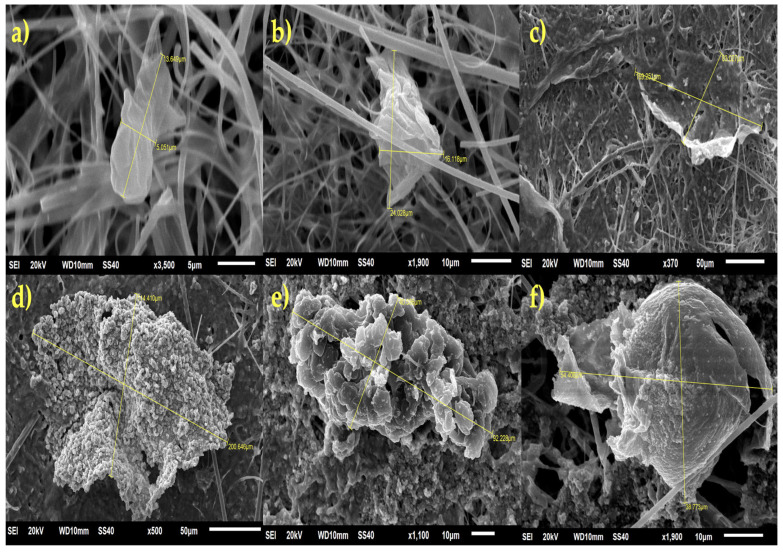
SEM images of MPs identified in the present study; (**a**) Long, sheet-like MP with a smooth surface and distinct shape; (**b**) angular and irregular MP with moderate roughness and distinct edges; (**c**) lamellar MP that is fractured and has an uneven surface and fractures; (**d**) globular agglomerated MP that has a high level of roughness; (**e**) lamellar/platelet aggregate MP that has a layered surface and appears rough; (**f**) hemispherical MP that has a dense, cavity-like surface.

According to the SEM analysis, it can be seen that MPs observed in Figure 6 can be classified as spherical, fragmented, fibrillar, or irregularly angular, which indicates that they were generated by various causes, including abrasion and/or mechanical breaking. It should be noted that the presence of linear stretch marks may be an indication of mechanical friction against internal system components (such as infusion pumps, filters, and tubulars) during administration or transportation due to the texture of the surfaces (i.e., soft, rough, microporosities, and linear stretch marks).

Adsorption of biological contaminants, proteins, or medications may be facilitated by superficial porosity. Processing and storage conditions, such as friction in peristaltic pumps, UV light (i.e., photooxidative deterioration), and the presence of plasticizing chemicals, may also be contributing factors. The same packing or additives used may be the source of spherical shapes. Its irregular shape, angular edges, and fractures indicate that the polymers (such as tubulatures, filters, and syringes) have been mechanically worn, particularly under low tension or by repetitive bending. MPs that are filamentous or fibrillar, with varying lengths and extremely thin diameters, may originate from medication bags or from the wear of fibre-reinforced plastic connectors. Laminar MP fragments, which resemble thin, flat plaques with parallel stretch lines occasionally, are a sign of the separation of thin film or laminate layers, like those seen in multilayer containers. Microbeads or recently formed MP fragments without extended friction exposure may be linked to smooth and polished surfaces. Its bioreactivity may be weaker due to decreased endothelial cell contact and biomolecule adsorption caused by a smaller effective surface area. The specific surface area may be greatly increased by rough or microporous surfaces, which may favour the opsonization (adsorption of plasma proteins) and promote the formation of biofilms or the collection of macrophages. Additionally, the roughness illustrated in some MPs observed in the SEM images indicates that the polymeric matrix was damaged by exposure to degrading mechanisms (such as photodegradation and mechanical stress) (Figure 6). Parallel lines, sometimes known as stretch marks, or grooves indicate continuous sliding against a stiff surface, such as a valve or peristaltic pump. Estimates of the direction and force exerted, as well as the number of equipment-use cycles, can be made using the mean and depth of the stretch marks.

To put it briefly, the SEM images of the MPs analyzed in the current study unequivocally demonstrate a mechanical wear origin. During transportation or administration, plastic components may experience friction and flexing, as indicated by stretch marks and angular fragmentation. It would be advised to determine how many pump and tubulatory cycles there are before they are required to be replaced. Photochemical degradation should also be considered, as it is clear that storage places (such as transparent bottles) have uneven porosity and surface cracks from UV light exposure. It is advisable to assess the exposure time of the IV bags and lighting conditions before applying them, particularly for Ecuador’s IV-MDs. Lastly, the presence of microfibers or mp laminars indicates surface or air pollution in the setup area. Implementing controls during the manufacturing process and working in sterile rooms with laminar flow are crucial for lowering the occurrence.

According to the International Council for Harmonization of Technical Requirements for Pharmaceuticals for Human Use (ICH) guidelines, Ecuador specifically falls under zone IVb (hot/higher humidity), meaning that stability conditions must be 30 °C ± 2 °C and 75% relative humidity (RH) ± 5%. This climate has an impact on the storage and functionality of IV-MD plastic materials across a large portion of Ecuador, particularly in the coastal and Amazonian regions. MPs and NPs in IV drugs must be taken into account by the National Agency for Health Regulation, Control, and Surveillance (ARCSA). Ecuador imports a lot of its medical supplies, which are made of plastics that might break down and spew particles. MP release into the infusion liquid is increased by the faster breakdown of polymers (such as PVC and PP) at these high temperatures and humidity levels. Recent studies suggest the length of the infusion, temperature, and UV light exposure all considerably boost the quantity of MPs administered to the patient. PVC-MPs were quantitatively measured to be considerably released when the infusion temperature was raised. With prolonged infusion periods and higher temperatures, these values rose even further [30]. Mechanical stress and friction on the plastic may trigger the rate to increase from 0.90 MPs/mL to 1.57 MPs/mL under infusion pump scenarios [43]. Hydrolysis of plastic additives has been favoured in high RH scenarios. It might weaken the material matrix thereby making it easier for MP fragments to be released. When MPs come into contact with saline fluids, this temperature-induced synergy encourages an increased MPs breakdown. The high levels of UV radiation in Ecuador are still another crucial consideration. When the MP-PP in infusion bags is exposed to heat and UV radiation, for instance, it becomes brittle and can release up to tens of thousands of MPs per bag (7500 MPs/bag in saline solutions); this quantity can triple after extended or surgical treatments [20]. Bags and tubes should be stored away from UV light sources and extreme heat in countries like Ecuador. The presence of MPs from the packaging and additives in hypertonic solutions (i.e., 3% NaCl) indicates that the chemical composition of the fluid also impacts particle release. Furthermore, the same study observed that the first 10–12 mL of fluid going through the tubing had the highest concentration of MPs; hence, removing the first volume can lower the initial pollutant load [33]. The zone IVb designation for Ecuador suggests environmental factors that promote MP release in IV infusion MDs and the deterioration of plastic components. High humidity, heat, and UV exposure in the country hasten this process, raising the possibility of giving the sufferer MPs.

National controls would need to be established at points of entry (customs, distributors) to evaluate purity and safety because a significant amount of the IV solutions used in Ecuador are imported and have limited traceability of their particle content. It is also necessary to establish a nationwide network of laboratories that are authorized to do advanced quality testing on intravenous drugs. Preventive measures such as climate control, filtration, initial volume disposal, and periodic equipment replacement are necessary to reduce this source of contamination and preserve patient health.

In this regard, creating awareness campaigns that support the safe use of medical supplies and encourage clinical procedures that are more cognizant of the potential of MP/NP contamination would be crucial for all the nations taken into consideration.

### 3.2. Polymer Hazard Index (PHI)

The MP concentration in glass IV-MDs for the Italian Brand ranged from 9 to 19 MPs/L, according to the data collected and analyzed for each IV-MD. In Spain, the glass IV-MDs had 13–20 MPs/L and the plastic IV-MDs had 211–240 MPs/L. All IV-MDs in Ecuador were made of plastic, and the MP concentrations they contained varied from 168 to 299 MPs/L. An instrument for evaluating the possible risk of polymers used in MD, such as IV-MDs, is the Polymer Hazard Index (PHI). A number of organizations (including the Food and Drug Administration–FDA, ISO, and ECHA) have suggested comparable approaches, despite the fact that there is not a single, globally accepted methodology. IV-MDs are regulated by a number of international standards, including FDA Guidance on Biocompatibility (ISO 10993-1, use of materials in contact with IV fluids), UNE-EN ISO 14971:2020 (risk management for MD), ECHA REACH (Classification of Hazardous Substances), and UNE-EN ISO 10993-1:2021 (Biological evaluation of IV-MDs).

The PHI in MD permits the estimation of a potential hazard of the polymer based on its chemical composition (i.e., monomers, plasticizers, additives, etc.), its ability to release harmful substances (leaching or migration), the toxicity of these substances (carcinogenicity, mutagenicity, reproductive toxicity, etc.), and the type and duration of contact with the human body. In order to determine the PHI from the data in the present study, the PHI formula needs to be modified according to the kind and quantity of polymer found per litre in IV-MD solutions. Consequently, the following simplified formula (Equation (1)) could be generated:

(1) $$PHI=\frac{1}{n}\sum \left(N_{i}\;\times \;H_{i}\right)$$

where *N_i_* is the number of MPs/L of polymer I, *H_i_* is the hazard of polymer *i* (these values are determined by toxicological classification or estimated risk, with one indicating a low degree of hazard and four indicating a very high one), and *n* is the total number of MPs examined. The *H_i_* values of each MP analyzed in the present study are summarized in Table 2.

For each brand and type of IV-MD, the exposure to MPs concentrations (MPs/L) and the PHI could be combined to generate a clinical risk matrix (Table 3) using the Hi values provided in Table 2.

**Figure 7:**
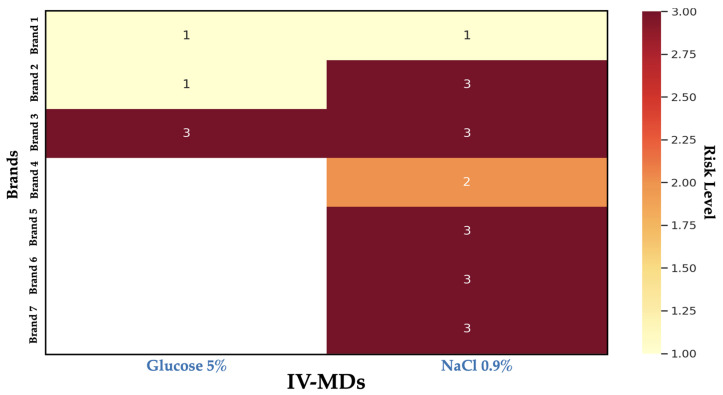
Heatmap of the clinical risk matrix.

The PHI stays at 1.7 despite significant variations in the amount of MPs/L. This is due to the index being weighted by the kind of polymer rather than its quantity. Due to the low proportion of the most hazardous polymers (such PU and SBR, 10% each one) and the low risk of the most prevalent ones (i.e., PE, PP), the PHI represents medium risk. The risk profiles of Brands 1, 2, and 4 were acceptable (low risk, low exposure). Due to their considerable exposure, Brands 3 and 6 should be closely watched and/or compared to other alternatives (moderate risk). Lastly, Brands 2 (NaCl 0.9%), 3 (NaCl 0.9%), 5, and 7 should be used with caution, particularly if they are used for long-term therapy (high risk) or in patients who are already at risk. To provide a clear and comparative perspective of the possible danger associated with the presence of MPs in various brands of IV-MD solutions, a heatmap of the clinical risk matrix was also created (Figure 7).

MP levels are generally higher in 0.9% NaCl solutions than in 5% glucose solutions. This could be due to different manufacturing or filling procedures, more contact time with plastics, or different chemical reactivity with the container. The exposure (MPs/L) has a considerable impact on the clinical risk level, but the PHI stays constant at 1.7. This demonstrates that the danger is mostly determined by the amount of polymer that migrates into the solution rather than just the type of polymer. The heatmap is a reference for choosing safer brands for clinical usage, ranking brands for biocompatibility testing or toxicological investigations, and arguing in front of regulatory bodies (i.e., FDA) for the necessity of MP limits in IV-MDs. The creation of patient safety-focused regulatory requirements, well-informed clinical judgments, and sample prioritization for toxicological investigations are all made easier by this visual tool.

### 3.3. Possible Risks in Human Health

The risk of MPs entering the bloodstream through IV injection is contingent upon a number of variables, including the kind of polymer, its size, shape, chemical additives, and its capacity to interact with tissues and cells. MPs in blood have not yet been thoroughly studied for their clinical toxicity; however, there have been reports of inflammatory reactions, increased cytokines (TNF-α, IL-1β), and NP accumulation in sensitive tissues (immune system, retina) [16,31]. The size of MPs < 10 µm, which allows them to permeate cell membranes and bioaccumulate in organs like the liver, spleen, brain, etc., is one of several elements that raise the risk to human health [16]. A further factor is the shape of MPs; MP fibres might result in chronic inflammation and are more challenging for phagocytes to remove. Furthermore, the potential inclusion of chemical additives (such as dyes, plasticizers, etc.) is hazardous in and of itself; yet, because of their surface charges or porosity, MPs may also serve as carriers of contaminants (such as heavy metals). Oxidative stress (OS), DNA damage, chronic inflammation, organ bioaccumulation, and interactions with endothelium and blood cells are among the potential biological effects in patients [48,49].

Microinflammation and macrophage activation are promoted by MPs, which are easily retained in peripheral capillaries and range in size from 10 to 50 µm. Long fibrous MPs (>100 µm) can mechanically clog tiny circulatory system branches. Furthermore, it is necessary to take into consideration migratory additives such phenols, phosphites, and UV stabilizers. When the MP enters toward contact with blood, these compounds may ascend to its surface and be released [48,49].

Every patient has a different risk of being exposed to MPs from IV-MDs (i.e., IV bags, tubing, and catheters). It is contingent upon the length of time and frequency of contact with plastic materials, the nature of the disease and its management, and the capacity of the body to remove toxins and foreign substances. For example, sharp PTFE fragments < 50 µm have the potential to cause local microthrombosis or harm vascular endothelium. Chronic inflammation may ensue from the long-term retention of any injected particles caused by biopersistence of the MP-PTFE. The release of residual diisocyanates from hydrolytically broken PU fragments carries the risk of cytotoxicity and sensitization. MP-PUs < 5 µm can be phagocytosed and cause the production of proinflammatory cytokines and other immunological reactions.

The specific risk in dialysis patients with chronic kidney disease (CKD) is an intriguing method of evaluating the risk of MPs on human health. Due to their constant contact with vascular access catheters, IV bags, dialysis filters, and blood lines, hemodialysis patients are especially vulnerable to polymeric materials. Due to their restricted renal excretion capacity, patients can accumulate MPs [50,51]. Patients receiving hemodialysis are extremely vulnerable because of their constant exposure (3–4 times per week, 3–5 h per session). Tubes, filters, catheters, and IV solution bags are among the various plastic lines they employ. MPs and toxins are not effectively eliminated by the kidneys. The immune system, liver, and reproductive system of the patient may all be impacted by certain MPs. Patients with chronic renal illness are particularly at risk for low-grade systemic inflammation, which can be brought on by ongoing contact with MPs.

As a result, the use of safer substitute materials should be promoted, and water and dialysis solution quality should be continuously checked. The main polymers found in medical equipment such infusion bags, tubes, and catheters are compared in Table 4, along with the possible risk level and associated factors for dialysis patients [4,52,53].

The urgent necessity to assess the toxicokinetics of MPs/NPs has been emphasized by the World Health Organization (WHO) and the European Food Safety Authority (EFSA). The quality of the materials, the usage conditions (temperature, contact time, and pH), and the national or regional laws governing medical supplies all affect the precise amounts of particle emission in hospital environments.

Due to their need for frequent infusions, central venous catheters (CVCs), and occasionally extracorporeal membrane oxygenation (ECMO), as well as their exposure to plastic materials in crucial conditions, patients with severe cardiovascular disease (CVD) are also at high risk for MPs. The risk of MPs is especially extremely high for newborns in neonatal intensive care units (NICUs). They are extremely susceptible to endocrine disruptors like DEHP, have a very high body surface area-to-exposure ratio, and are constantly on feeding tubes, catheters, ventilation, and IV bags.

**Figure 8:**
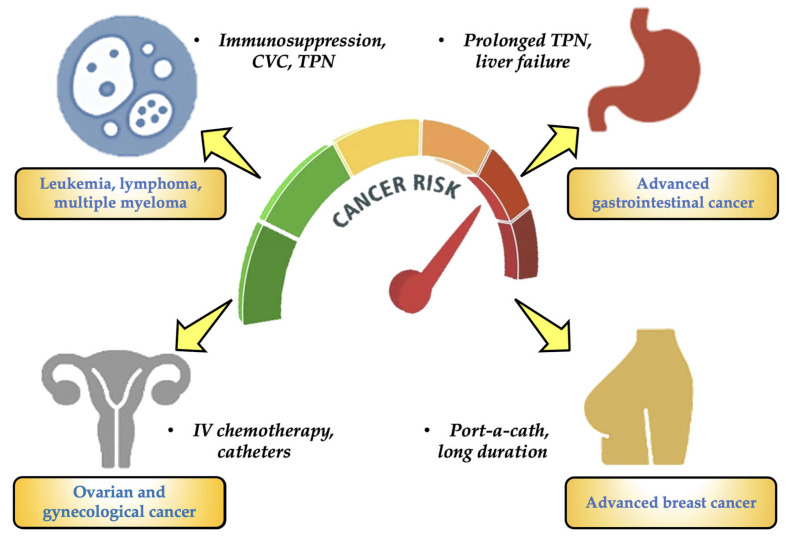
The main types of cancer with a high-risk rate of MPs.

The length and frequency of cancer treatment, the type of therapy (chemotherapy, parenteral nutrition, etc.), the effects of the cancer on the liver, kidney, and immune system, and the type of vascular access (central venous catheter, port-a-cath, etc.) all influence the risk of MP exposure in cancer patients. The following lists the cancer forms that, given the frequent use of IV bags, may be more susceptible to MP exposure. Figure 8 shows the four main tumours that have the highest risk of MPs.

- Patients with blood cancers (such as leukemias, lymphomas, and multiple myeloma): They have received protracted (months to years) treatment with several cycles of IV chemotherapy and are at the highest risk for MPs in oncology. They also continue to employ port-a-catheters, peripherally inserted central catheters (PICCs), CVCs, and other devices. These patients also have severely weakened immune systems and often need IV antibiotics, transfusions, and parenteral nourishment [54,55].
- Advanced gastrointestinal cancer (liver, pancreas, colon, and stomach): It is another type of cancer that has a significant risk of MPs. Long-term TPN is necessary for many patients, and they are frequently exposed to plastic IV bags, which frequently contain lipid emulsions that can encourage the leaching of chemicals like DEHP. This particular type of patient also has compromised intestine or liver function, which lowers the removal of toxins [56,57].
- Gynecological and ovarian carcinomas: For this kind of cancer, the risk for MPs is considerable. This particular type of cancer requires intensive IV chemotherapy treatments. Catheters, continuous fluids, and peritoneal lavage are commonly employed. Patients may require TPN as a result of ascites and malabsorption events [58,59].
- Advanced breast cancer: It is the final stage of a high-risk cancer. Patients receive chemotherapy and IV targeted treatments for an extended period of time. Some people have been using port-a-catheters for years. Patients are exposed to medical plastics on a regular basis, but their functional status is typically better than that of patients with the previously described cancer categories [57,60].

Furthermore, lipophilic IV solution formulations (such as TPN and lipid-soluble chemotherapies) might encourage the release of plasticizers. Cancers that affect the liver or kidneys are less able to get rid of harmful chemicals and MPs. Exposure to plastics in the environment or while receiving medical treatment may exacerbate symptoms that certain patients may have that resemble those of chronic chemical poisoning, such as exhaustion and immunological dysfunction.

### 3.4. Putative Limitations of This Study

Not all commercially available IV-MDs were selected for this study; only the most common IV-MDs from Ecuador, Spain, and Italy were analyzed. They were all from the same batch of IV-MDs. This study highlights the value of early investigation and the necessity of more extensive IV-MD research in the future. The experiments standardized manufacturing time and storage techniques by detecting tiny particles (MPs) under typical IV-MD circumstances. Temperature and humidity levels were also maintained to regulate other physicochemical factors. It is crucial to keep in the forefront that any of the previously described elements may have an impact on MP development. Future studies should therefore look into additional variables that can affect MP generation in IV-MDs, such as differences in manufacturing timeframes.

Fourier transform infrared spectroscopy (μ-FTIR) has not yet been used to identify MPs produced during the infusion procedure. In the present study, we have chosen to chemically characterize the MPs using this technique for the following reasons:

- An accurate analytical method that clarifies molecular chemical bonds and offers comprehensive details on polymer types and their functional groups, μ-FTIR is essential for MP identification [31].
- Its ability to identify polymers containing polar functional groups, including hydroxyl (O–H) and carbonyl (C=O), gives it a distinct edge when analyzing particular MP categories.
- Since FTIR sample preparation is simple, the analysis is more thorough and efficient. Rapid examination of several samples is made possible by the measuring method’s exceptional speed, which is particularly useful in high-throughput situations like material characterization and environmental monitoring. Additionally, it reduces fluorescence and signals produced by pollutants, additives, pigments, and other materials [31].

In general, the detection of MPs released during the infusion process using μ-FTIR can offer a more thorough insight into the true amounts of small particles entering the human body. Using μ-FTIR, this study seeks to identify and describe MPs in actual infusion operations. Additionally, it attempts to replicate the infusion procedure in order to examine the effectiveness of various filter devices when used in conjunction with the infusion apparatus for the removal of MPs.

Future research could identify smaller particles and use complementing techniques like high-resolution Raman spectroscopy (also known as atomic force microscopy-Raman (AFM-Raman) to obtain an even more exact characterization. In vivo studies to assess the biological effects of MPs in humans have not yet been carried out; however, the current study detects and describes the existence of MPs in IV-MDs. Consequently, conclusions about possible pathophysiological effects must be regarded as provisional and based in extrapolations from previous studies.

## 4. Future Perspectives and Potential Solutions

Possible solutions and future opportunities might include a programme to revise hospital policies to incorporate the monitoring of MPs in infusions (IV-MDs), educating medical staff members on proper handling and storage techniques for plastics, encouraging research into substitutes for reduced-release materials, and conducting clinical analyses of MP exposure. Globally standardized procedures should be established to track the amounts of MPs and NPs in pharmaceutical-grade items. Strict temperature and humidity control in preparation and storage spaces (preferably <25 °C) and the storage of bags and tubes in opaque cabinets to block UV light, particularly in countries like Ecuador, are further measures to reduce the risk of MPs/NPs. The first 10–12 mL of IV fluid should be thrown away before connecting to the patient, according to the outcomes of previous studies presented in the literature [33,61]. Using 0.2 µm filters in the infusion circuit to keep MPs and some NPs would be an additional method. Lastly, to reduce the continual release of particles, the length of time that tubing and catheters are used could be adjusted by replacing them every 24 to 48 h. Additionally, encourage the incorporation of MPs/NPs risk assessments into drug approval procedures and pharmacopeias. Establish precise limits for MPs and NPs in parenteral goods, just like you would for endotoxins or particles. To evaluate the effect on human health, extensive toxicokinetic and immunological studies are required. Epidemiological studies would be required for monitoring the outcomes of patients exposed to MPs/Ns through IV treatment in order to conduct public health surveillance and patient education. Regarding chemicals limits, we encourage the inclusion of MP/NP criteria/limitations in pharmacopeial monographs and suggest a pilot study/trial to evaluate the relationship between MP load in infusions and endothelial activation biomarkers (i.e., circulating VCAM-1, IL-6).

## 5. Conclusions

IV-MDs from three distinct countries were compared for the first time. The 29 IV-MD samples included MPS, with concentrations ranging from 9 to 20 MPs/L in glass containers and 166 to 299 MPS/L in plastic bags. Due to heat, moisture, UV rays, and extended supply chains, Ecuador’s IV-MDs displayed the highest MPs concentrations. Compared to plastic bags and catheters, the use of glass containers greatly decreased MP contamination. While the average PHI remains at 1.7, marks with more than 200 MPs/L were considered a high clinical risk. Cell barriers can be crossed by the IV infusion of MPs < 10 µm, which can lead to oxidative damage, inflammation, and potential bioaccumulation. As a preventative precaution, the first 10–12 mL of IV infusion should be avoided. Afterwards, 0.2 µm filters should be used, along with UV light and temperature control (<25 °C). Similarly to endotoxins, MPs limits must be set in pharmacopoeic monographs and established monitoring systems immediately. To assess long-term effects, high-resolution characterization studies, in vivo toxicity trials, and epidemiological surveillance are recommended.

## Figures and Tables

**Table 1:** Main segmentations of the European IV-MDs market.

By Type of Solution	By Final User	By Geographical Division
Total Parenteral Nutrition (TPN): Complete treatments that give patients who are unable to obtain enteral or oral feeding all the nutrients they need. In 2023, this market category dominated the European market, and through 2030, it is anticipated to continue growing at the quickest rate. Peripheral Parenteral Nutrition (PPN): Used for patients who need temporary or partial nutritional support. Crystalloids: Aqueous electrolyte solutions, including 5% glucose and saline (0.9% NaCl), are frequently used to maintain electrolyte balance and hydrate the body. Colloids: High-molecular-weight molecule-containing solutions that are utilized to increase plasma volume in hypovolemic shock and other conditions. Others: Contains customized solutions for particular situations and blood products.	Hospitals: The main users of IV-MDs, particularly in operating rooms and critical care units. Clinics: For simple procedures and outpatient treatments, use IV-MDs. Home Care: This market is expanding as more IV treatments are given at home, such as to palliative care patients or those with chronic illnesses.	Germany: Due to its ageing population and sophisticated healthcare system, this country leads the European market for IV-MDs. France and the United Kingdom: Important markets that prioritize efficiency and innovation in the administration of IV therapy. Italy and Spain: Expanding markets where IV-MDs are being used more often in a range of clinical situations.

**Table 2:** Main *H_i_* values of the MPs analyzed.

MPs	*H_i_* *	Distribution (%)
PE	1	25
PP	1	20
PET	2	15
PA	2	10
SBR	3	10
PU	3	10
PTFE	2	5
EVA	1	5

* Hazard estimation based on persistence, toxicity, leaching potential, and additives.

**Table 3:** Clinical risk matrix.

Brand	IV-MD	MPs/L (Mean ± SD)	PHI Value	PHI Level	Exposition Level	Clinical Risk
Brand 1	Glucose 5%	12 ± 4	1.7	Medium	Low	Low
Brand 1	NaCl 0.9%	16 ± 3	1.7	Medium	Low	Low
Brand 2	Glucose 5%	17 ± 4	1.7	Medium	Low	Low
Brand 2	NaCl 0.9%	227 ± 15	1.7	Medium	High	High
Brand 3	Glucose 5%	185 ± 17	1.7	Medium	High	High
Brand 3	NaCl 0.9%	219 ± 22	1.7	Medium	High	High
Brand 4	NaCl 0.9%	22 ± 6	1.7	Medium	Moderate	Moderate
Brand 5	NaCl 0.9%	259 ± 57	1.7	Medium	High	High
Brand 6	NaCl 0.9%	191 ± 42	1.7	Medium	High	High
Brand 7	NaCl 0.9%	240 ± 37	1.7	Medium	High	High

**Table 4:** Risk assessment of main MP types present in IV-MDs, according to the literature and in our study.

MP Type	Potential Risk	Reasoning
PVC	High	Contains harmful substances that can cause chlorine release, like phthalates (i.e., plasticizer), which are endocrine disruptors
PS	High	It is easily fragmented into NPs, can induce oxidative stress, cell damage, inflammation
SBR	Moderate-High	Can leach residual monomers (styrene, butadiene) and additives (antioxidants, accelerators); prone to oxidative degradation into NPs, triggering oxidative stress, inflammation, and potential cytotoxicity
PET	Moderate	It can release Sb (i.e., used in its production) associated with oxidative stress and intracellular accumulation
PU	Moderate	It can degrade, releasing toxic diisocyanates and cause inflammation
PA	Moderate	Less investigated, but it might have inflammatory and physical impacts
EVA	Moderate	Contains vinyl acetate units that may leach monomer (a probable carcinogen) and additives; prone to mechanical fragmentation into NPs, potentially inducing oxidative stress and inflammatory responses
PP	Low-moderate	Generally thought to be inert, they may trigger immunological reactions if they break apart into NPs
PE	Low-moderate	Chemically inert, whereas cumulative and physical effects are not completely ruled out, just like PP
PTFE	Unknow potential	Extremely resistant to chemicals, but capable of releasing some harmful fluorinated compounds

## Data Availability

The datasets used and/or analyzed during this study are available from the corresponding author on reasonable request.

## Supplementary Materials

The following supporting information can be downloaded at: https://www.mdpi.com/article/10.3390/toxics13070597/s1, Table S1: The 29 IV-MDs of seven different brands analyzed in the present study; Figure S1: Main types of cancer in Ecuador, Spain, and Italy; Table S2: Main MP characteristics of IV-MDs analyzed (in triplicate); Table S3. Comparative analysis of MPs found in IV-MDs.

## Abbreviations

The following abbreviations are used in this manuscript:

ABS	Acrylonitrile Butadiene Styrene
AFM-Raman	Atomic Force Microscopy-Raman
ARCSA	Agencia Nacional de Regulación, control y Vigilancia Sanitaria
CAGR	Compound Annual Growth Rate
CKD	Chronic Kidney Disease
CVC	Central Venous Catheter
CVD	Cardiovascular Diseases
DEHP	Di(2-ethylhexyl)phthalate
ECIS	European Cancer Information System
ECMO	Extracorporeal Membrane Oxygenation
EFSA	European Food Safety Authority
EPS	Expanded Polystyrene
EVA	Ethylene Vinyl Acetate
FDA	Food Drug of Administration
FTIR	Fourier Transform Infrared Spectroscopy
GLP	Good Field and Laboratory Practices
ICH	International Council for Harmonization of Technical Requirements for Pharmaceuticals for Human Use
IV	Intravenous Administration
IV-MDs	Intravenous Medical Devices
LDPE	Low Density Polyethylene
MDs	Medical Devices
MPs	Microplastics
NPs	Nanoplastics
OPEs	Organophosphate Esters
OS	Oxidative stress
PA	Polyamide
PAHs	Polycyclic Aromatic Hydrocarbons
PCBs	Polychlorinated Biphenyls
PE	Polyethylene
PTFE	Polytetrafluoroethylene (Teflon)
PET	Polyethylene Terephthalate
PHI	Polymer Hazard Index
PICC	Peripherally Inserted Central Catheter
PM	Particulate Matter
PMMA	Polymethylmethacrylate
POPs	Persistent Organic Pollutants
PP	Polypropylene
PPN	Peripheral Parenteral Nutrition
PS	Polystyrene
PVA	Polyvinyl Alcohol
PVC	Polyvinyl Chloride
PU	Polyurethane
RH	Relative Humidity
ROS	Reactive Oxygen Species
SBR	Styrene-Butadiene
SEM	Scanning Electron Microscopy
TDCPP	Tris(1,3-dichloro-2-propyl)phosphate
TPN	Total Parenteral Nutrition
UV	Ultraviolet Light
WHO	World Health Organization
μFTIR	Micro Fourier Transform Infrared Spectroscopy
