# Supplementary material for: Health Risks from Microplastics in Intravenous Infusions: Evidence from Italy, Spain, and Ecuador
Source: Toxics. 2025 Jul 16;13(7):597. doi: 10.3390/toxics13070597 (PMC12298248; doi:10.3390/toxics13070597)
Supplement: Supplementary file 1 [file toxics-13-00597-s001.zip › toxics-3712744-supplementary.pdf]

Supporting Materials

Health Risks from Microplastics in Intravenous Infusions: Evidence from Italy, Spain, and Ecuador

Table S1. The 29 IV-DMs of seven different brands analyzed in the present study

ITALY			
**Brand**	**Type of IV-MDs**	**Volume (mL)**	**Type of packaging**
Brand 1	NaCl 0.9%	500	Glass Bottle
Brand 1	NaCl 0.9%	500	Glass Bottle
Brand 1	NaCl 0.9%	500	Glass Bottle
Brand 1	Glucose 5%	500	Glass Bottle
Brand 1	Glucose 5%	500	Glass Bottle
Brand 1	Glucose 5%	500	Glass Bottle
**SPAIN**			
**Brand**	**Type of IV-MDs**	**Volume (mL)**	**Type of packaging**
Brand 2	Glucose 5%	500	Glass Bottle
Brand 2	Glucose 5%	500	Glass Bottle
Brand 2	Glucose 5%	500	Glass Bottle
Brand 2	NaCl 0.9%	500	Plastic Bag
Brand 2	NaCl 0.9%	500	Plastic Bag
Brand 2	NaCl 0.9%	500	Plastic Bag
Brand 3	NaCl 0.9%	100	Plastic Bag
Brand 3	NaCl 0.9%	100	Plastic Bag
Brand 3	NaCl 0.9%	100	Plastic Bag
Brand 3	Glucose 5%	500	Plastic Bag
Brand 3	Glucose 5%	500	Plastic Bag
Brand 3	Glucose 5%	500	Plastic Bag
**ECUADOR**			
**Brand**	**Type of IV-MDs**	**Volume (mL)**	**Type of packaging**
Brand 4	NaCl 0.9%	1,000	Plastic Bottle
Brand 4	NaCl 0.9%	1,000	Plastic Bottle
Brand 4	NaCl 0.9%	1,000	Plastic Bottle
Brand 5	NaCl 0.9%	1,000	Plastic Bag
Brand 5	NaCl 0.9%	1,000	Plastic Bag
Brand 6	NaCl 0.9%	1,000	Plastic Bag
Brand 6	NaCl 0.9%	1,000	Plastic Bag
Brand 6	NaCl 0.9%	1,000	Plastic Bag
Brand 7	NaCl 0.9%	1,000	Plastic Bag
Brand 7	NaCl 0.9%	1,000	Plastic Bag
Brand 7	NaCl 0.9%	1,000	Plastic Bag

Figure S1. Main types of cancer in Ecuador, Spain, and Italy

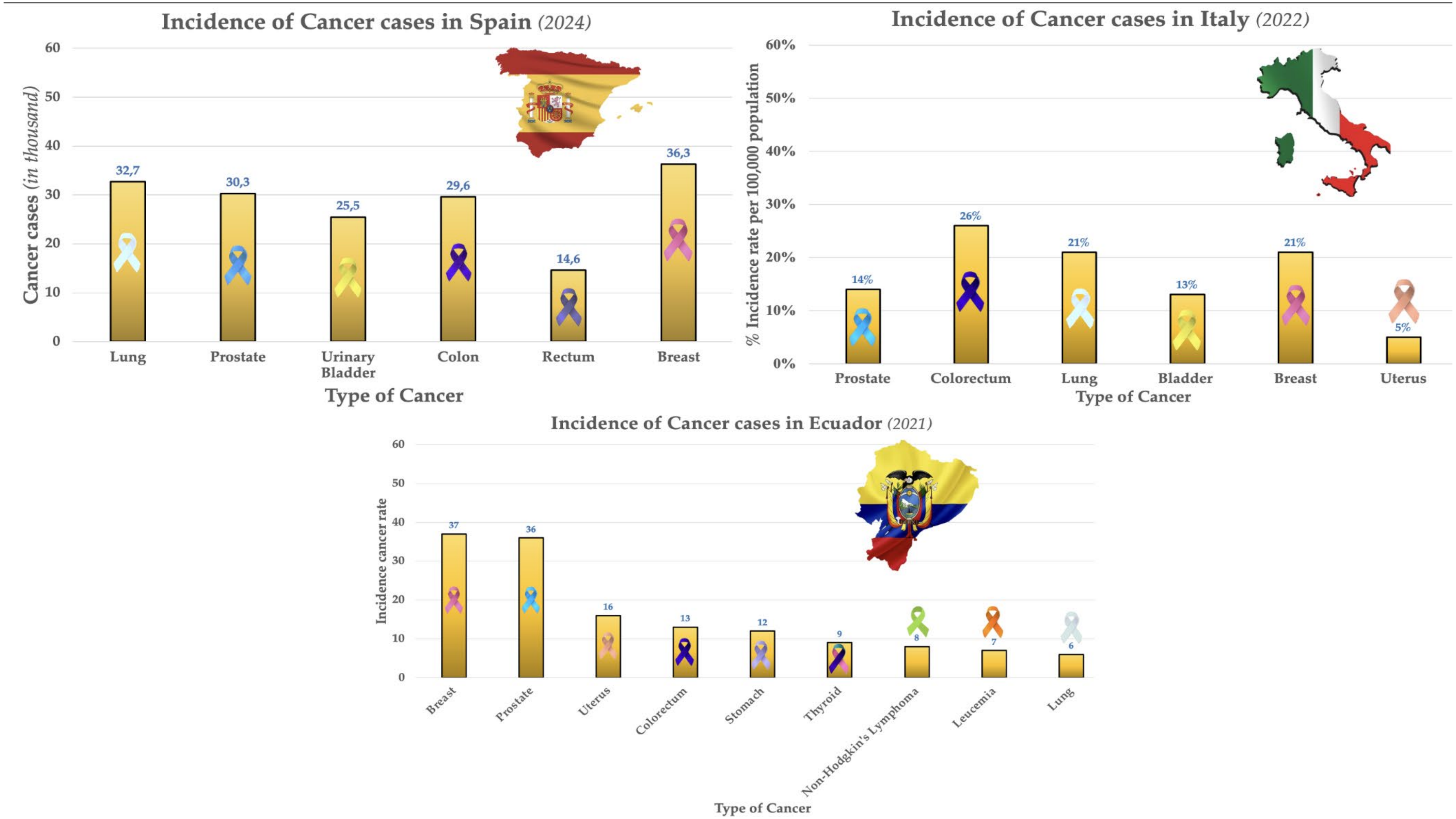

Table S2. Main MP characteristics of IV-MDs analyzed (*in triplicate*)

ITALY

Brand	IV-infusion	Packaging	MPs/L	% MP Shape		% MP Size (μm)				
				% Fibres	% Fragments	1 - 50	51 - 100	101 - 250	251 - 500	> 500
Brand 1	NaCl 0.9%	Glass Bottle	15	35	65	39	28	15	10	8
Brand 1	NaCl 0.9%	Glass Bottle	13	50	50	35	25	19	14	7
Brand 1	NaCl 0.9%	Glass Bottle	19	31	69	44	22	15	15	4
Brand 1	Glucose 5%	Glass Bottle	11	40	60	46	21	17	8	8
Brand 1	Glucose 5%	Glass Bottle	9	44	56	52	20	11	10	7
Brand 1	Glucose 5%	Glass Bottle	16	39	61	43	22	16	12	7

SPAIN

Brand	IV-infusion	Packaging	MPs/L	% MP Shape		% MP Size (μm)				
				% Fibres	% Fragments	1 - 50	51 - 100	101 - 250	251 - 500	> 500
Brand 2	Glucose 5%	Glass Bottle	17	41	59	44	20	8	16	12
Brand 2	Glucose 5%	Glass Bottle	13	32	68	43	18	22	13	4
Brand 2	Glucose 5%	Glass Bottle	20	35	65	50	12	20	15	3
Brand 2	NaCl 0.9%	Plastic Bag	211	48	52	30	24	19	21	6
Brand 2	NaCl 0.9%	Plastic Bag	230	50	50	33	16	39	-	12
Brand 2	NaCl 0.9%	Plastic Bag	240	43	57	37	21	30	5	7

Table 2. *Continued*

Brand 3	NaCl 0.9%	Plastic Bag	196	37	63	39	24	20	11	6
Brand 3	NaCl 0.9%	Plastic Bag	222	44	54	34	26	22	7	11
Brand 3	NaCl 0.9%	Plastic Bag	240	39	61	31	24	18	16	11
Brand 3	Glucose 5%	Plastic Bag	167	35	65	36	20	24	14	6
Brand 3	Glucose 5%	Plastic Bag	188	40	60	34	19	21	17	9
Brand 3	Glucose 5%	Plastic Bag	201	37	63	38	27	18	14	3
**ECUADOR**										
**Brand**	**IV-infusion**	**Packaging**	**MPs/L**	**% MP Shape**		**% MP Size (μm)**				
				**% Fibres**	**% Fragments**	**1 - 50**	**51 - 100**	**101 - 250**	**251 - 500**	**> 500**
Brand 4	NaCl 0.9%	Plastic Bottle	219	41	59	56	24	9	7	4
Brand 4	NaCl 0.9%	Plastic Bottle	208	38	62	51	21	8	11	9
Brand 4	NaCl 0.9%	Plastic Bottle	234	40	60	46	27	11	8	8
Brand 5	NaCl 0.9%	Plastic Bag	299	33	67	32	23	21	24	-
Brand 5	NaCl 0.9%	Plastic Bag	219	39	61	39	22	16	16	7
Brand 6	NaCl 0.9%	Plastic Bag	168	44	56	34	25	35	6	-
Brand 6	NaCl 0.9%	Plastic Bag	239	32	68	39	20	24	6	11
Brand 6	NaCl 0.9%	Plastic Bag	166	46	54	31	24	19	14	12
Brand 7	NaCl 0.9%	Plastic Bag	255	35	65	37	28	20	11	4

Table 2. *Continued*

Brand 7	NaCl 0.9%	Plastic Bag	198	30	70	32	27	15	20	6
Brand 7	NaCl 0.9%	Plastic Bag	266	33	67	46	27	18	-	9

Table S3. Comparative analysis of MPs found in IV-MDs

**Polymer properties**	**PE**	**PP**	**EVA**	**PU**	**PA/Nylon**	**PET**	**SBR**	**PTFE**
Biocompatibility	High	High	High	Very High	High	High	Moderate	Very High
Flexibility	Medium	Low	High	Very High	Medium	Low	High	Very low
Transparency	Good	Good	Excellent	Good	Regular	Excellent	-	-
Chemical resistance	High	High	Medium-High	Very High	High	Very High	Medium	Excellent
Autoclave safety (121°C)	No	Yes	No	Some	Yes	Yes (limited)	No	Yes
Gamma Ray resistance	High	High	High	Good	High	Variable	Low	High
Lipid compatibility	High	High	Excellent	Excellent	Good	Excellent	Low	Excellent
Main use in IV system	Bags, tubes	Connectors, caps	Bags, tubes	Catheters, pressure tubes	Connectors, clamps	Multi-layer barriers	Plungers, seals	Special catheters
Cost	Low	Low	Medium	Medium	Medium	Medium	Low	High
